# Bilateral dacryoadenitis in adult-onset Still’s disease: A case report

**DOI:** 10.1515/biol-2022-0472

**Published:** 2022-12-09

**Authors:** Qing Huang, Weimin He

**Affiliations:** Department of Ophthalmology, West China Hospital of Sichuan University, Chengdu, 610041, Sichuan, China

**Keywords:** adult-onset Still’s disease, dacryoadenitis, lacrimal gland

## Abstract

We present an unusual case of bilateral dacryoadenitis in a middle-aged patient with adult-onset Still’s disease (AOSD). We reviewed relevant clinical studies addressing the association between lacrimal lesions and AOSD. A 50-year-old Chinese woman with a 4 year history of recurrent fever and rashes was admitted to the hospital. She had also developed nodules on both eyelids 10 months before admission. After undergoing resection of the left lacrimal gland, the patient received steroids and immunosuppressive therapy. The patient showed good postoperative recovery during the 20 month follow-up. In this case, the pathological examination conducted after orbital surgery helped clinicians differentiate between dacryoadenitis and other orbital lesions. In a review of the literature, dacryoadenitis occurred after the onset of AOSD, and all cases showed non-granulomatous chronic inflammation by histopathology, which indicated that the lacrimal gland may be an inflammatory target and is affected by systemic inflammation in AOSD.

## 1 Background

Adult-onset Still’s disease (AOSD) is a rare systemic inflammatory disorder of unknown etiology, characterized by recurrent high-spiking fever, an evanescent salmon pink rash, arthralgia, and multiorgan involvement [1]. Other common manifestations include myalgia, liver abnormalities (hepatomegaly and abnormal liver biochemistry), pleuritis, pericarditis, and splenomegaly are seen less commonly [2]. AOSD sometimes involves ophthalmic manifestations, such as uveitis, keratitis, ptosis, oculomotor disorders, erythema, chemosis, and orbital pseudotumor [3]. Dacryoadenitis has also been linked to AOSD, the most common form of idiopathic orbital inflammation. However, little is known about lacrimal gland involvement in AOSD patients.

Here we report a middle-aged Chinese woman diagnosed with AOSD who developed bilateral lacrimal gland lesions. We also review and summarize relevant literature on dacryoadenitis in AOSD patients with the aim of improving our understanding of this disease.

## 2 Case presentation

A 50-year-old Chinese woman was admitted to our hospital with complaints of recurrent high fever, rash, and joint pains in the 4 years prior to admission. She had also developed nodules on both eyelids in the 10 months prior to hospitalization.

**Figure 1:**
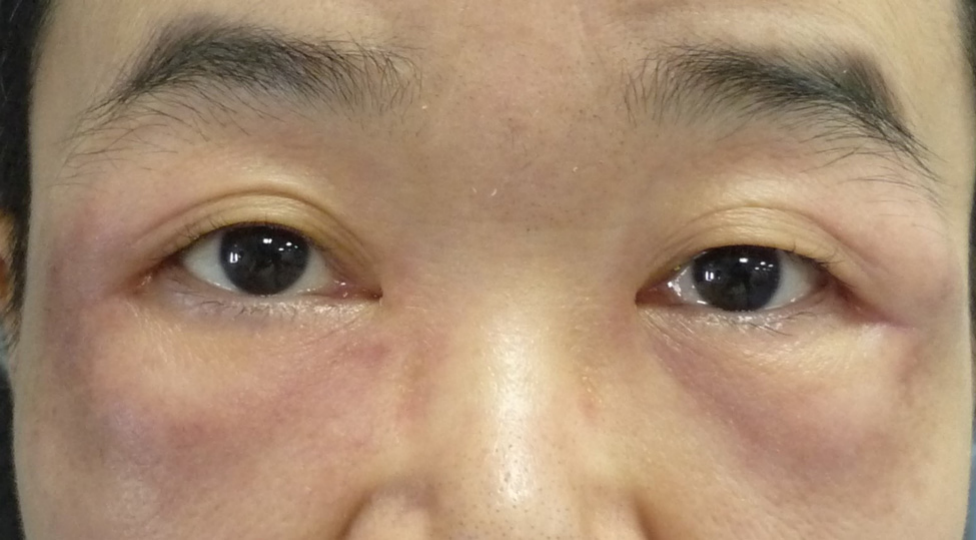
Preoperative image of the external eye in our patient at admission to our hospital.

**Figure 2:**
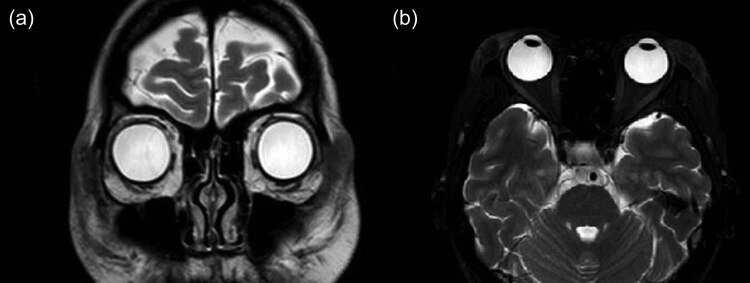
Contrast enhanced T2-weighted orbital MRI, showing thickened soft tissue around the orbit and enlargement of both lacrimal glands. (a) Coronal slice. (b) Horizontal slice.

Her physical examination showed maculopapular eruption on the back of her hands, upper limbs, neck, and back, with enlarged superficial lymph nodes in the neck, axilla, and inguen. Laboratory tests revealed a white blood cell count of 14,020 per mm^3^, neutrophil percentage in granulocytes of 92.3%, and C-reactive protein level of 19.80 mg/L, with negative rheumatoid factor and antinuclear antibody tests. Visual acuity was measured to be 20/25 in the right eye and 20/100 in the left eye. Palpation showed the distinct presence of nodules on both eyelids (Figure 1). Although the anterior segment and fundus were normal, enhanced magnetic resonance imaging (MRI) of the eye showed thickened soft tissue around the orbit with heterogeneous enhancement (Figure 2). Based on the clinical symptoms and MRI scans, the patient was diagnosed as AOSD with dacryoadenitis.

**Figure 3:**
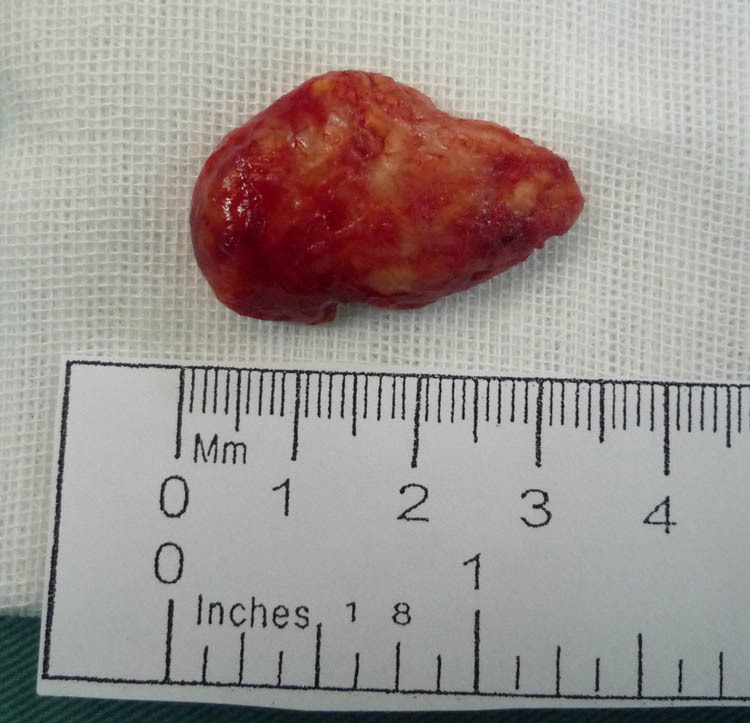
Image of the excised orbital mass. The cut section of the specimen appears solid and tan-white.

**Figure 4:**
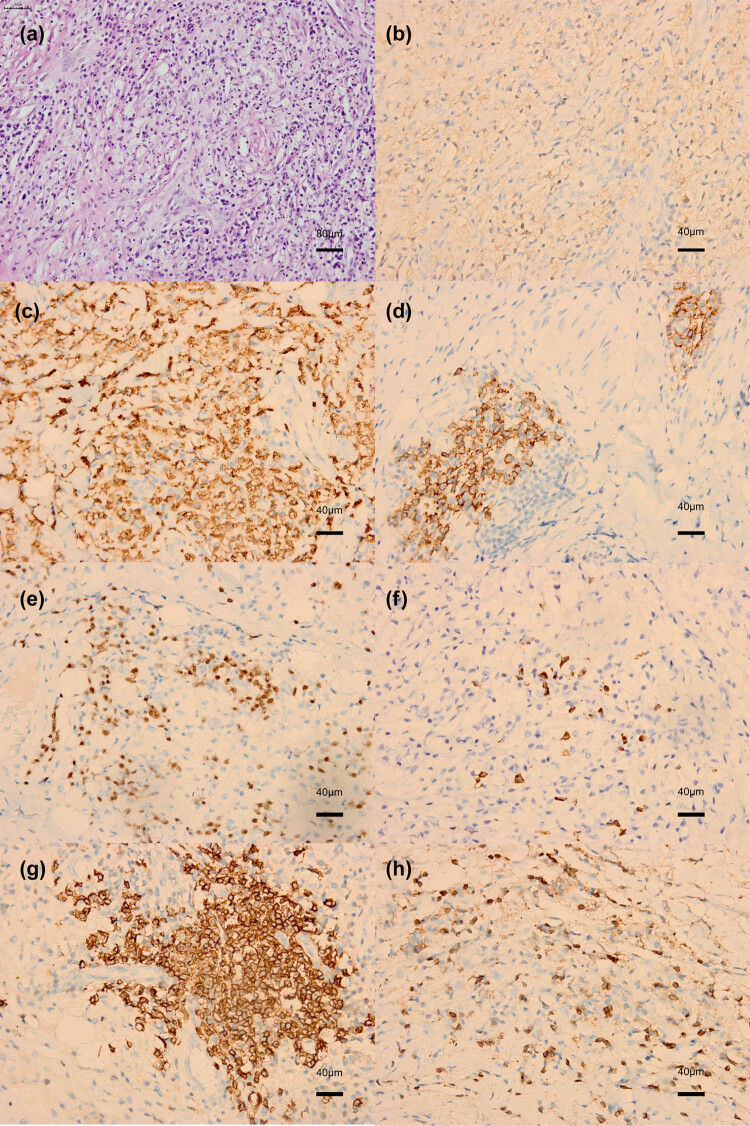
Histopathological examination of the excised mass. (a) Histopathologic section stained with hematoxylin-eosin (H&E, magnification ×200), showing foam cell granuloma with lymphocytes and plasma cell infiltration. (b) Positive staining for CD163 (magnification ×400). (c) Positive staining for PGMS (magnification ×400). (d) Positive staining for plasma cell CD138 (magnification ×400). (e) Positive staining for MUM1 (magnification ×400). (f) Sparse positive staining for IgG4 (magnification ×400). (g) Sparse positive staining for CD20 in lymph cells (magnification ×400). (h) Sparse positive staining for CD3 (magnification ×400).

The patient underwent surgery for resection of the left orbital mass. During surgery, we observed a superior lateral lacrimal and orbital mass that adhered closely to the orbital periosteum and subcutaneous tissue. The excised mass was solid and tan-white in color (Figure 3). Pathological examination showed foam cell granuloma with lymphocytes and plasma cell infiltration. The biopsy stained positive for CD163, propylene glycol mannate sulfate (PGMS), plasma cell CD138, multiple myeloma oncogene 1 (MUM1), IgG4 (sparse positive, 16/high-power field), lymph cell CD20 (partially positive), and CD3 (partially positive) (Figure 4).

**Figure 5:**
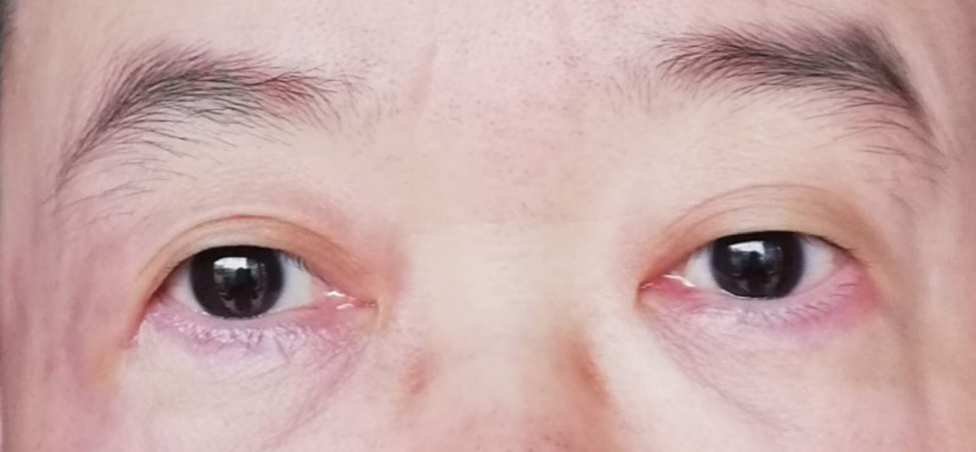
Postoperative image of the external eye in our patient with AOSD at 20 months after surgery.


After undergoing surgery, methylprednisolone and cyclosporine were given for 20 months, during which she showed good postoperative recovery, with resolution of eyelid nodules (Figure 5). The fever, joint symptoms, and skin rash were also relieved.

**Informed consent:** Informed consent has been obtained from all individuals included in this study.
**Ethical approval:** The research related to human use has been complied with all the relevant national regulations, institutional policies and in accordance with the tenets of the Helsinki Declaration, and has been approved by the authors’ institutional review board or equivalent committee.

## 3 Discussion

AOSD is a multisystemic autoinflammatory disorder of unknown etiology. It was first described in 1971 based on the similarity between clinical symptoms presented by 14 adults and those observed in children with Still’s disease [4]. There are only two previous reports of dacryoadenitis occurring with AOSD. Our patient developed bilateral dacryoadenitis after systemic presentation of AOSD. Considering the Yamaguchi classification criteria [5], our case met all the major criteria: fever of at least 39°C, intermittent, lasting 1 week or longer; arthralgias, lasting 2 weeks or longer; typical rash; leukocytosis (>10,000 per mm^3^), with 80% or more granulocytes; and met two minor criteria: lymphadenopathy and negative rheumatoid factor and antinuclear antibody. After undergoing resection surgery, and receiving steroid and immunosuppressive therapy, the patient showed good postoperative recovery. Orbital surgery and pathophysiology helped our understanding of lacrimal lesions complicated by AOSD.

Our review of the literature identified 2 reports of dacryoadenitis in women with AOSD [6,7], involving 2 Japanese, 1 French, and 1 Chinese woman who ranged in age from 26 to 62 years (mean age 45.8 ± 15 years; Table 1). In all four patients, dacryoadenitis occurred after the onset of AOSD: two developed symptoms in both eyes, and the others developed symptoms in only one eye. All four patients showed non-granulomatous chronic inflammation by histopathology, which indicated that the lacrimal gland may be an inflammatory target and is affected by systemic inflammation in AOSD. The immunohistochemical staining was helpful to differentiate from infectious disease, xanthoma, and Rosai-Dorfman disease. After resection, these patients received either steroid or interleukin-1 (IL-1) inhibitor therapy. All patients showed good postoperative recovery (Table 1). Unlike previously reported patients, serum ferritin level in our patient was not elevated, may be due to the long course of disease, and previous intermittent treatment with steroids before admission [8].

**Table 1:** Case reports of dacryoadenitis in patients with AOSD

	Case report A [6]		Case report B [7]	Present report
Name of 1^st^ author, Year	Bannai, 2015		Breillat, 2018	Qing, 2021
Country	Japan		France	China
Age (years)	26	62	45	50
Sex	Female	Female	Female	Female
Orbital location	Bilateral	Left	Left	Bilateral
MRI	Circumscribed, enhancing mass in the bilateral eyelid consistent with the lacrimal gland extending to the anterior temporal side through the soft orbital tissue	Left lacrimal gland and periorbital soft tissue swelling with strong contrast enhancement extending into the temporal occipitofrontalis muscles, rectus superior muscles, and the enthesis of the levator palpebrae superioris muscles	Diffuse enlargement of the left lacrimal gland and periorbital tissue manifesting as hyperintensity on T2-weighted images. This enlargement was markedly enhanced after gadolinium injection on T1-weighted images	Thickened soft tissue around the orbit with heterogeneous enhancement
Histology	Lacrimal gland infiltrated with mild lymphoid cells around the small vessels and fibroconnective tissue consistent with mild chronic inflammation	Lymphoplasmacytic infiltration and fibrosis	Superficial, perivascular, and interstitial dermal polymorphic infiltrate containing neutrophils, few lymphocytes, little atypia, and no plasma cells or sclerosis	Foam cell granuloma with lymphocytes and plasma cell infiltration
Treatment	Prednisolone, tocilizumab	Prednisolone	IL-1 inhibitor	Methylprednisolone, cyclosporine
Follow up (months), status	Unknown, resolution	Unknown, resolution	5, Resolution	20, Resolution

Clinicians often find it difficult to diagnose and treat AOSD because of the wide range of differential diagnoses and clinical manifestations associated with it [9]. Current therapies for AOSD include nonsteroidal anti-inflammatory drugs (NSAIDs), glucocorticoids, anti-rheumatic drugs, polyvalent intravenous immunoglobulins, IL-1 receptor antagonists (anakinra), and anti-human IL-6 monoclonal antibodies [1,2,9]. NSAIDs often fail to control the symptoms of AOSD and many patients experience adverse events; corticosteroid therapy is considered the first-line treatment, but dependency occurs in approximately 45% of cases. Methotrexate can reduce the daily corticosteroid intake, but remission speed is slow, and it can cause severe adverse events, including liver toxicity and pneumonitis. Biologic drugs can be administered in cases refractory to the above treatments, but it is expensive for patients in developing countries [10,11]. To date, the treatment of AOSD has relied on empirical data from prospective or retrospective studies. Double-blinded randomized trials with suitable sample sizes are required to develop effective treatments for patients with AOSD.

## 4 Conclusion

We report a rare case of bilateral dacryoadenitis in a middle-aged Chinese woman with AOSD. This diagnosis was supported by pathological examination after lacrimal resection, which helped exclude other orbital diseases. The histopathological findings of non-granulomatous chronic inflammation indicated that the lacrimal gland may be an inflammatory target and is affected by systemic inflammation in AOSD. Our case highlights the need for long-term follow-up to prevent recurrence of dacryoadenitis. Future research must focus on ophthalmic manifestations to gain a better understanding of the diagnosis and treatment of patients with AOSD. Additionally, the development of practical serological or molecular diagnostic marker(s) that could be utilized for the early diagnosis of atypical bilateral dacryoadenitis is recommended.

## References

[1] Bagnari V, Colina M, Ciancio G, Govoni M, Trotta F. Adult-onset Still’s disease. Rheumatol Int. 2010;30(7):855–62. 10.1007/s00296-009-1291-y 20020138

[2] Efthimiou P, Paik PK, Bielory L. Diagnosis and management of adult onset Still’s disease. Ann Rheum Dis. 2006;65(5):564–72. 10.1136/ard.2005.042143 PMC1798146 16219707

[3] Cush JJ, Leibowitz IH, Friedman SA. Adult-onset Still’s disease and inflammatory orbital pseudotumor. N Y State J Med. 1985;85(3):110–1. 3857478

[4] Bywaters EG. Still’s disease in the adult. Ann Rheum Dis. 1971;30(2):121–33. 10.1136/ard.30.2.121 PMC1005739 5315135

[5] Yamaguchi M, Ohta A, Tsunematsu T, Kasukawa R, Mizushima Y, Kashiwagi H, et al. Preliminary criteria for classification of adult Still’s disease. J Rheumatol. 1992;19(3):424–30. 1578458

[6] Bannai E, Yamashita H, Takahashi Y, Tsuchiya H, Mimori A. Two cases of adult-onset Still’s disease with orbital inflammatory lesions originating from the lacrimal gland. Intern Med. 2015;54(20):2671–4. 10.2169/internalmedicine.54.4838 26466709

[7] Breillat P, Tourte M, Romero P, Hayem G, Padovano I, Costantino F, et al. Interleukin-1 inhibitors and dacryoadenitis in adult-onset Still’s disease. Ann Intern Med. 2018;168(6):455–6. 10.7326/L17-0401 29230477

[8] Maranini B, Ciancio G, Govoni M. Adult-onset Still’s disease: Novel biomarkers of specific subsets, disease activity, and relapsing forms. Int J Mol Sci. 2021;22(24):13320. 10.3390/ijms222413320 PMC8706484 34948117

[9] Gerfaud-Valentin M, Maucort-Boulch D, Hot A, Iwaz J, Ninet J, Durieu I, et al. Adult-onset Still’s disease: Manifestations, treatment, outcome, and prognostic factors in 57 patients. Med (Baltim). 2014;93(2):91–9. 10.1097/MD.0000000000000021 PMC4616309 24646465

[10] Giacomelli R, Ruscitti P, Shoenfeld Y. A comprehensive review on adult-onset Still’s disease. J Autoimmun. 2018;93:24–36. 10.1016/j.jaut.2018.07.018 30077425

[11] Efthimiou P, Kontzias A, Hur P, Rodha K, Ramakrishna GS, Nakasato P. Adult-onset Still’s disease in focus: Clinical manifestations, diagnosis, treatment, and unmet needs in the era of targeted therapies. Semin Arthritis Rheum. 2021;51(4):858–74. 10.1016/j.semarthrit.2021.06.004 34175791

